# Temporal and Spatial Dynamics of Different Interictal Epileptic Discharges: A Time-Frequency EEG Approach in Pediatric Focal Refractory Epilepsy

**DOI:** 10.3389/fneur.2020.00941

**Published:** 2020-09-08

**Authors:** Younes Jabran, Mahdi Mahmoudzadeh, Nicolas Martinez, Claire Heberlé, Fabrice Wallois, Emilie Bourel-Ponchel

**Affiliations:** ^1^INSERM UMR 1105, Research Group on Multimodal Analysis of Brain Function, University of Picardie Jules Verne, Amiens, France; ^2^INSERM UMR 1105, Pediatric Neurophysiology Unit, Amiens University Hospital, Amiens, France

**Keywords:** time-frequency analysis, interictal epileptic spikes, electrical source imaging, high-density EEG, refractory focal epilepsy

## Abstract

**Objective:** Characterization of the spatial and temporal dynamics of interictal epileptic discharges (IED) using time-frequency analysis (TFA) and electrical-source localization (ESL).

**Methods:** TFA was performed on IED (spikes, spike waves, and polyspike waves) recorded by high-density-EEG (HD-EEG) in 19 refractory focal epileptic children. Temporal modulations related to IEDs were analyzed in a time window around the IED peaks [−1,000 to 1,000 ms]. Spatial modulations were analyzed by ESL in the time-frequency and time domains.

**Results:** IED were associated with complex power spectral modulations. We observed increases in power spectrum (IPS) patterns specific to IED type. For spikes, the TFA pattern consisted of an IPS (−100 to +100 ms, 4–50 Hz). For spike waves, the IPS was followed by a second IPS (+100 to +400 ms, 4–10 Hz), corresponding to the slow wave. IPS patterns were preceded (−400 to −100 ms, 4–40 Hz), and followed (+100 to +400 ms) by a decrease in the power spectrum (DPS) (*n* = 8). For 14 out of 19 patients, at least one ESL method was concordant with the epileptogenic area. For the remaining five patients, all of them had temporal epilepsies. ESL in the time-frequency domain (DPS/IPS) provided concordant (*n* = 6) or complementary (*n* = 4) information to the ESL in the time domain concerning the epileptogenic zone. ESL in time-frequency domain (DPS/IPS) was the only method to provide concordant information concerning the epileptogenic zone in three patients.

**Significance:** TFA demonstrates complex time-frequency modulations of the neuronal networks around IED, suggesting that the pathological mechanisms are initiated well before onset of the classical hyper-synchronization of the IED. Combining time and time-frequency analysis of the ESL provides complementary information to define the epileptogenic zone in refractory focal epilepsy.

## Highlights

- Time-frequency analysis reveals specific power spectral EEG modulations depending on IED type analyzed.
- Time-frequency analysis reveals a decrease in spectral power in neuronal activity, occurring ±400 ms before and after the IED.
- Combining time and time-frequency analysis of the ESL provides complementary information to define the epileptogenic zone.

## Introduction

Epilepsy affects 50 million people worldwide (1) and is a major public health issue. Despite the availability of new antiepileptic drugs (2), the prevalence of refractory focal epilepsy (RFE) has remained stable, accounting for 33% of epileptic patients (3). A better knowledge of the pathophysiological mechanisms underlying epileptic processes, notably in the epileptogenic network, is necessary to improve the management of patients with RFE.

Interictal epileptic discharges (IEDs), spikes, spike waves, polyspike waves, are the hallmark of the epileptogenicity of the neuronal network during the interictal state (4). IEDs are classically related to increasing activity of a large synchronized population of neurons. Nevertheless, the mechanisms that propel neurons to IEDs are more complex, associating synaptic and non-synaptic interactions (4), and we have also demonstrated changes in the cellular configuration (5, 6) and hemodynamic environment (7, 8). At the synaptic level, the complexity of the neuronal interactions have been addressed by multi-unit activity recordings in presurgical stereotaxic EEG (S-EEG), which demonstrates interactions of multiple distinct neuronal populations (9), with (i) various firing patterns (either increased, decreased, or no modulation, according to the population of neurons) and (ii) specific temporal relationships with the IEDs (9).

High-density electroencephalography (HD-EEG) can be analyzed in the time and time-frequency domains to non-invasively assess the complexity of the neurophysiological mechanisms associated with IEDs. Time-frequency analysis (TFA) can characterize frequency modulations temporally related to IEDs in spatially distinct neuronal populations. Indeed, frequency modulations have been observed at the network level from −400 to +400 ms around the IED peaks by TFA, independently of the epilepsy type (7, 9, 10). Similar time-frequency modulations have also been observed in the epileptic rat model around the interictal epileptic spikes and is therefore not species-specific (5). Nevertheless, the complex spatial and temporal dynamics specific to the different types of IEDs (spikes, spike waves, polyspike waves) have been less studied. We applied the TFA approach, previously developed by our laboratory (5, 6, 10), to a population of children with RFE presenting different types of IEDs to better characterize the specificity of TFA modulations according to the type of IEDs and of the type of epilepsy.

## Materials and Methods

### Population

Nineteen children aged between 0 and 16 years (mean age: 9.9 years, age range from 3 months to 14 years), with refractory mono-focal epilepsy (3, 11) and IEDs by HD-EEG were included in this study, conducted in the Amiens University Hospital Department of Pediatric Clinical Neurophysiology (Table 1). The epileptogenic sub-lobar area was defined according to the usual clinical assessment criteria based on clinical data, ictal and inter-ictal scalp video-EEG, neuroimaging results (MRI-CT), metabolic data (FDG-PET scan), and stereotactic EEG results and post-surgical outcome when available (Table 1).

**Table 1:** Clinical characteristics of the RFE children population.

**Patients**	**Etiology**	**Seizure type**	**Clinical outcome (follow-up) Surgery**		**Epileptogenic sub-lobar area**	
1	Brain infection (empyema)	FIAS: Non motor (behavior arrest)	No	-	*Right*	Pref (DL-DM)
2	Brain infection (meningo-encephalitis)	FAS: non motor (cognitive)	Yes	Engel 1 (6 months)	*Left*	Oper-Ins Temp (MA-MP)
3	Cortical malformation (polymicrogyria)	FAS: motor (epileptic spasms + clonic)	No	-	*Left*	Pref (DL-VL-VM-DM)
4	Cortical malformation (DNET)	FAS: motor (epileptic spasms)	Yes	Engel 1 (5 years)	*Right*	Temp (LP)
5	Neonatal cerebral vascular ischemia	FIAS: Motor (clonic)	No	-	*Right*	Premot (L) Cent (L)
6	Cortical malformation (lissencephaly)	FIAS: Non-motor (behavior arrest)	No	-	*Right*	occ (L-M)
7	Cortical malformation (dysplasia)	FIAS: Motor (automatisms)	Yes	Engel 1 (4 years)	*Left*	Temp (MA)
8	Neonatal cerebral vascular ischemia	FIAS: Non-motor (autonomic)	Yes	Engel 1 (2 years)	*Left*	Pariet (L) Temp (LP-MP), Oper-Ins
9	Oligodendrioglioma	FIAS: Non-motor (behavior arrest)	Yes	Engel 1 (6 years)	*Right*	Temp (LA-MA)
10	N/A	FIAS: non- motor (sensory)	Yes	Engel 1 (1 year)	*Left*	Pariet (L-M)
11^*^	N/A	FIAS: motor (clonic)	-	-	*Left*	Pref (DL-VL-VM-DM)
12	Neonatal cerebral vascular ischemia	FIAS: motor (clonic)	No	-	*Left*	Pariet (L-P) Occ (L-M)
13	N/A	FIAS: Non-motor (sensory)	Yes	Engel 1 (3 years)	*Right*	Temp (LP) Occ (L)
14^*^	N/A	FAS: motor (Epileptic spasms + tonic)	-	-	*Right*	Temp (LP-MP) Pariet (M-L)
15	Brain infection (meningo-encephalitis)	FAS: Non-motor (sensory)	Yes	Engel 3 (4 years)	*Left*	Temp (MA-LA)
16	DNET	FAS: Non-motor (sensory)	Yes	Engel 1 (2 years)	*Left*	Pariet (L)
17	N/A	FAS: motor (automatisms)	Yes	Engel 2 (3 years)	*Right*	Occ (L)
18^*^	N/A	FIAS: motor (tonic)	No	-	*Right*	Pariet (L-M)
19	N/A	FAS: motor (automatisms)	No	-	*Left*	Temp (LA-MA)

*The epileptogenic sub-lobar area was defined according to the presurgical evaluation based on clinical, ictal/inter-ictal electroencephalography, and imaging characteristics*.

*Y, years; m, months; FIAS, focal impaired-awareness seizures; FAS, Focal Aware Seizures; R, right; N/A, not available; L, left; VM pref, ventral-medial prefrontal; DM pref, dorsal-medial prefrontal; VL pref, ventral-lateral prefrontal; DL pref, dorsal-lateral prefrontal; M premot, medial premotor; L premot, lateral premotor; M cent, medial central; L cent, lateral central; MA temp, medial-anterior temporal; LA temp, lateral-anterior temporal; MP temp, medial-posterior temporal; LP temp, lateral-posterior temporal; M pariet, medial parietal; L pariet, lateral parietal; M occ, medial occipital; L occ, lateral occipital; Oper-Ins, operculo-insular; and Junc, temporo-parieto-occipital junction*;

* *patients recorded using 128-channel EGI*.

The local ethics committee approved this study (CPP Nord-Ouest, No. A00782-39). Children and their parents provided written informed consent before inclusion.

### HD-EEG Acquisition

Sixteen patients were assessed by 64-channels HD-EEG (Ag/AgCl electrodes (EsayCap®), 10/10 international system (12) in DC mode, with a mastoid reference, at a sampling rate of 1,024 Hz, amplified by A.N.T® (Enschede, The Netherlands) or COMPUMEDICS® (Table 1).

Three additional patients (Table 1) were assessed by 128-channels HD-EEG (HydroCel™ Geodesic Sensor Net, EGI®) in DC mode with a Cz reference electrode at a sampling rate of 1,000 Hz. The electrode impedances were kept below 10 kΩ. HD-EEG recordings were performed during quiet arousal. During acquisition, synchronized video, deltoid muscle, heart rate, and respiratory activity were recorded.

HD-EEG acquisition lasted from 20 min to 2 h with 24 h delay from the last seizure.

### HD-EEG Analysis

**Figure 1:**
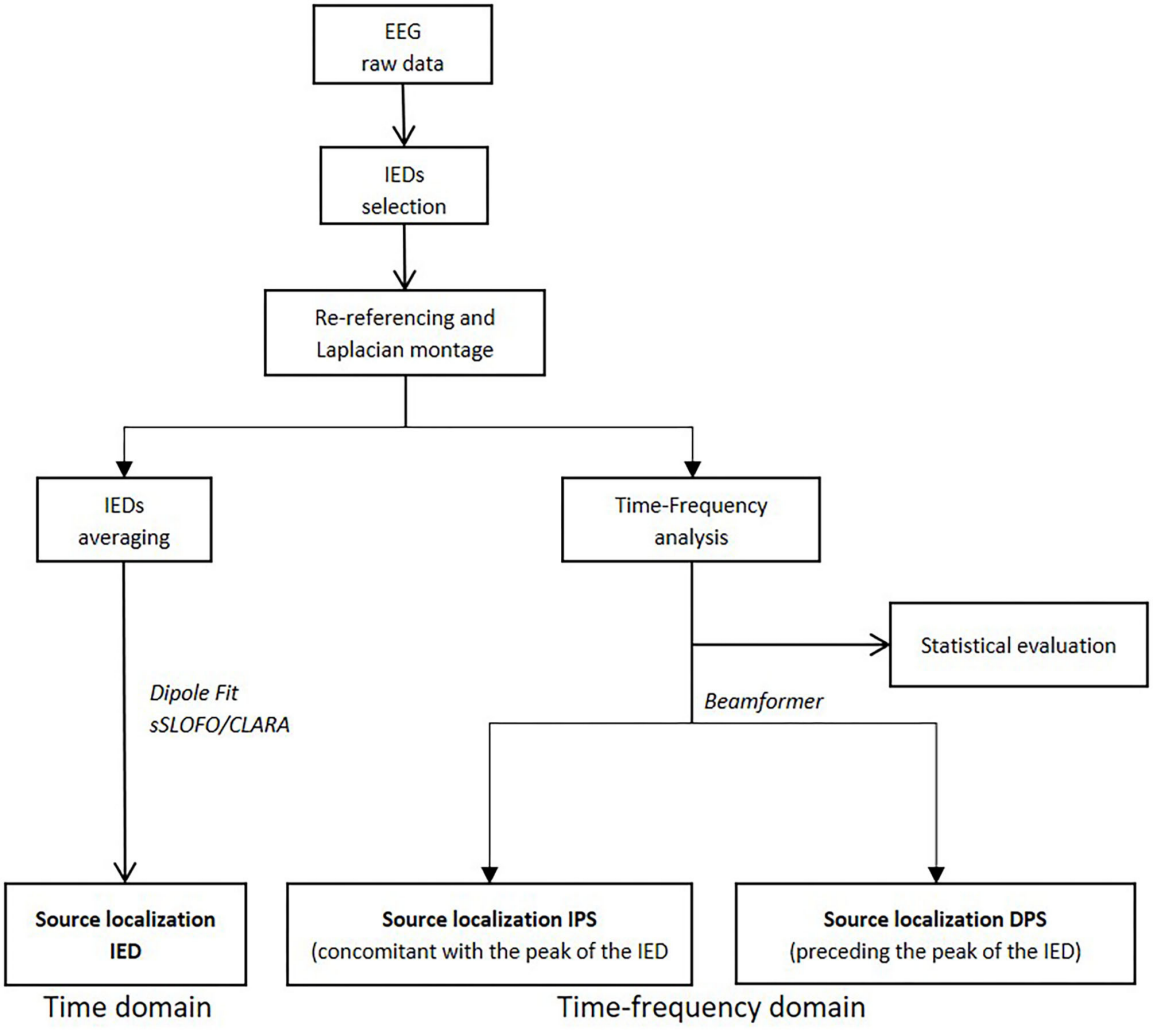
Diagram summarizing the various steps of the study (i.e., time-frequency analysis processing and source localization in time and time-frequency domains).

HD-EEG analysis was performed in three steps using BESA Research® (13): (a) IED selection, (b) TFA, and (c) ESL in the time and in time-frequency domains (Figure 1).

#### IED Selection

Manual selection of IEDs (spikes, spike waves, and polyspike waves) was first performed by one neurophysiologist (YJ) and then validated by a second neurophysiologist (FW or EBP). Only events confirmed by the two neurophysiologists were included in the analysis. Spike was defined as an isolated wave with a duration >80 ms and an amplitude above 50 μV (14). Spike-wave was defined as complex of two waves, associating a spike follows by a second wave with a duration of 200–500 ms and an amplitude above 50 μV (14). Poly-spike wave was defined as a spike and wave complex with more than one spike (14). For IEDs selection, an average reference montage was used. A bandpass of 0.5–70 Hz and a notch filter (50 Hz) were applied only for the IED-selection process. Visually identified artifacted channels (muscular, ocular, high impedances, etc.) were rejected by the neurophysiologists. Between 1 to 2 channels were rejected in six patients. For the other ones, all the channels were included in the study.

Isolated non-overlapping epochs were selected around the first negative IED peak (T0) ([−2,000 to 2,000 ms] around T0) Epochs which contained more than 1 IED in the time window considered ([−2,000 to 2,000 ms] around T0) were discarded from the analysis.

#### Time-Frequency Analysis (TFA)

For TFA, including high-frequency activity (4–200 Hz) (15), the signals were analyzed in a scalp surface Laplacian montage to reduce volume-conduction effects.

Non-overlapping epochs were selected around the first negative IED peak (T0) (−1,000 to 1,000 ms around T0). First, relative baseline segments lasting 500 ms (−1,000 to −500 ms before T0) were defined for each channel for each IED epoch. A second analysis was performed for each patient with a more remote baseline period (−2,000 to −1,500 ms before T0) to account for any possible baseline selection effect. In the second analysis, the window used for TFA corresponded to −2,000 to 2,000 ms around T0.

TFA was expressed as the relative power change to baseline activity at a time-frequency bin compared with the mean power over the baseline epoch for that frequency, $TFR=\frac{\text{P}\left(\text{t, f}\right)-{\text{P}}_{\text{baseline}}\left(\text{f}\right)}{{\text{P}}_{\text{baseline}}\left(\text{f}\right)}\cdot 100\%$ where *P*_(t, f)_ = the power at time t and frequency f and P_baseline_(f) = the mean activity at frequency f over the baseline epoch.

TFA was first computed for each selected IED epoch at each frequency using the complex demodulation method (16). TFA was extended to include all EEG channels to establish regional specificity. For each frequency of interest f0, the following three steps were performed. First, the original time-domain signal (i.e., not subjected to any offline filtering) was multiplied by sin(2πf0f) and cos(2πf0f), respectively. This modulation operation shifts every signal at frequency f to the difference and sum frequencies (f ± f0) in the frequency domain. Second, the resulting two signals were low-pass filtered to extract the frequency range originally centered around f0 and that was shifted to the low frequency range (f–f0). Thus, the low-pass cut-off frequency sets half of the width of the frequency band for which the envelope amplitude and phase is computed. Third, the two output signals of previous step define the real and imaginary part of a complex signal as a function of time. Its magnitude corresponds to half of the envelope amplitude. The time-frequency representation was calculated over each IES epoch. Frequencies were sampled (Gaussian filter) in 2 Hz steps and latencies were sampled in 25 ms steps, corresponding to a time-frequency resolution of ±2.83 Hz and ±39.4 ms at each time-frequency bin (full width at half maximum).

The probability that spectral power differs significantly from the average power during the baseline interval was investigated. Two-sided bootstrap testing was performed on the trials (17). z0 and the test statistics z^*^ for a given bootstrap sample were computed as

$$z0=\frac{{\bar{y}}_{2}-{\bar{y}}_{1}}{\sqrt{\frac{\sigma_{y_{2}}^{2}}{n}+\frac{\sigma_{y_{1}}^{2}}{n}}},z^{*}=\frac{{\bar{y}}_{2}^{*}-{\bar{y}}_{1}^{*}-({\bar{y}}_{2}-{\bar{y}}_{1})}{\sqrt{\frac{\sigma_{y_{2}}^{*}{\;}^{2}}{n}+\frac{\sigma_{y_{1}}^{*}{\;}^{2}}{n}}}$$

where

$$\begin{array}{l} {ȳ}_{1}=\frac{1}{n}\underset{trials}{\sum }{\bar{P}}_{baseline,i}\\ {ȳ}_{2}=\frac{1}{n}\underset{trials}{\sum }P_{i} \end{array}$$

Here, P denotes the power and n the number of trials. An asterisk denotes the value of a bootstrap sample. R bootstrap samples were computed. This computation was performed for each sampling point in the time-frequency space. The *p*-value was approximated from the number of bootstrap samples, where z^*2^ > ${\text{z}}_{0}^{2}$: *p* = (1 + # {z^*2^ > ${\text{z}}_{0}^{2}$})/(R + 1).

Correction for multiple testing was performed using the method of Simes et al. (18) to reduce the false-positive rate. It is applied to each IED epoch, which belongs to one frequency bin. This means that each channel and each frequency bin was treated as an independent measurement, whereas the statistical tests over the time series within one frequency bin were treated as multiple measurements (19). Each physiological phenomenon leads to different dependencies between the measurement channels. It was assumed that the activity which we wished to study is based on oscillatory phenomena, which were likely to be confounded to define the frequency bands. All *p*-values of one frequency bin and channel were sorted in ascending order (p_*i*_, *i* = *1,.,N*). The maximum index *m* in the sorted array, for which p_*i*_ < α^*^i/N, was determined. All values with *i* < *m* were accepted as significant detection. The significance level α was set to 0.05.

#### Control Conditions for TFA

The same procedure was performed using random triggers under physiological conditions. First, IED-free epochs (*n* = 30) were randomly selected during quiet arousal by experienced neurophysiologists (EBP-YJ-FW). T0 corresponds to the central point of the control segment [−1,000 to 1,000 ms]. Second, physiological vertex sharp waves (IED-free epochs) were selected. T0 corresponds to the maximum negative deflexion of vertex sharp waves [−1,000 to 1,000 ms].

#### Time and Time-Frequency Domain Electrical Source Localization (ESL)

ESL was performed in the time and time-frequency domains to determine the spatial distribution of the source of the IEDs.

ESL was resolved with standardized finite-element realistic head models according to the child's age (range of 2 years). The value of conductivity was 0.33 S.m^−1^ for the brain and the scalp, 0.1 S.m^−1^ for CSF and 0.0042 S.m^−1^ for the bone.

Eighteen sub-lobar regions were defined: ventral-medial prefrontal (VM pref), dorsal-medial prefrontal (DM pref), ventral-lateral prefrontal (VL pref), dorsal-lateral prefrontal (DL pref), medial premotor (M premot), lateral premotor (L premot), medial central (M cent), lateral central (L cent), medial-anterior temporal (MA temp), lateral-anterior temporal (LA temp), medial-posterior temporal (MP temp), lateral-posterior temporal (LP temp), medial parietal (M pariet), lateral parietal (L pariet), medial occipital (M occ), lateral occipital (L occ), operculo-insular (Oper-Ins), and temporo-parieto-occipital junction (Junc) (20).

##### Time-domain ESL

The selected IEDs were first averaged and source localization was then solved using a dipolar source model algorithm applied to the first negative peak of the IED (i.e., *dipole fit)* (20, 21) to localize the source in the time domain. For some patients, distributed methods (CLARA®, sSLOFO®) were also applied.

##### Time-frequency domain ESL

**Figure 2:**
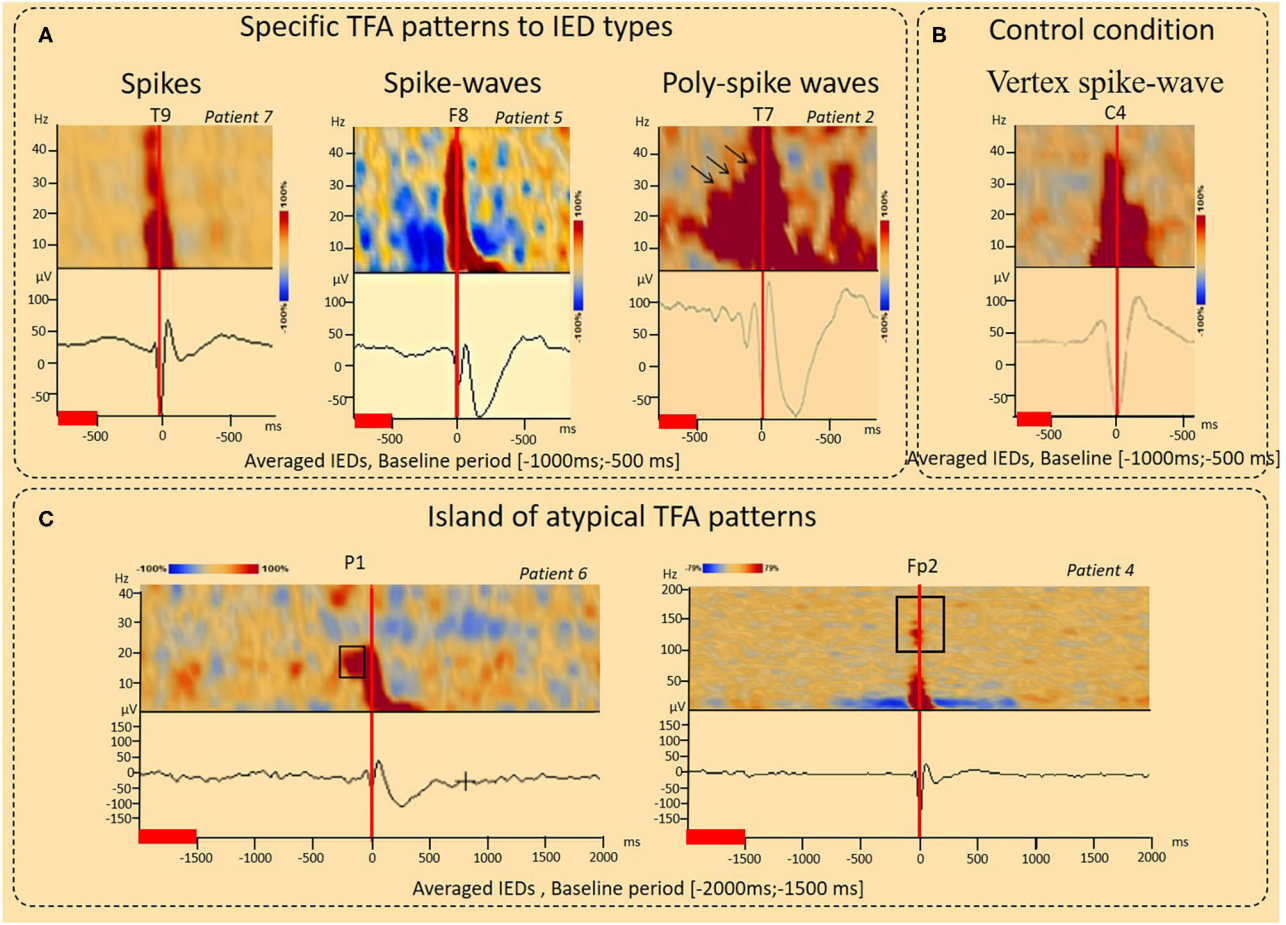
Results of the time-Frequency analysis (TFA) according to interictal epileptic discharge type and under control conditions. **(A)** Typical IPS patterns specific to IED type. For spikes, the TFA pattern consisted of a single IPS (−100 to 100 ms, 4–50 Hz, *Patient 17*). For spike-waves, this IPS was followed by a second IPS in the range of 4–10 Hz (100–400 ms), corresponding to the slow-wave, resulting in a “boot shape” (*Patient 5*). For polyspike waves (*Patient 2*), successive IPS, corresponding to the successive spikes were followed by a boot-shaped pattern concomitant with the slow-wave. A decrease in the power spectrum (DPS) symmetrically surrounded the spike-wave peaks and related IPS (−400 to 400 ms, 4–40 Hz) was observed (*patient 5*). **(B)** Control condition, vertex spike waves. An increase in spectral power concomitant to the vertex sharp wave in the time domain was observed (−100 to 200 ms, 4–40 Hz) without any other power spectral perturbations. **(C)** Island of atypical TFA patterns. IPS related to IEDs were preceded by a localized IPS pattern (pattern 1) (−200 to −100 ms, 15–25 Hz, *Patient 6*). IPS related to IEDs were associated with isolated IPS islands (pattern 2) (−100 to 100 ms, 100–150 Hz), likely corresponding to high-frequency oscillations (HFO) (*Patient 4*). The red lines corresponded to the baseline periods.

**Figure 3:**
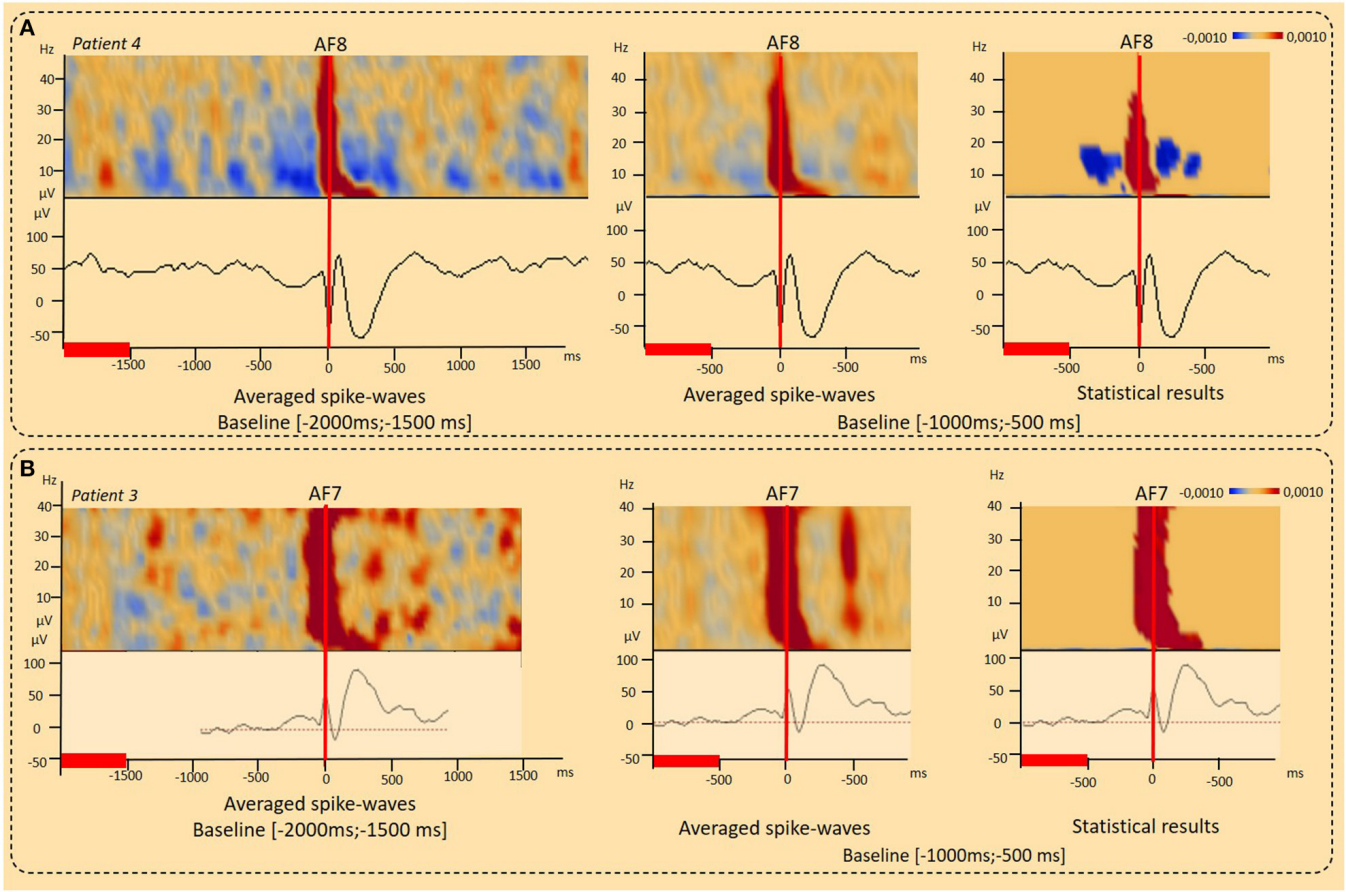
Results of the time-Frequency analysis (TFA) according to the baseline period considered. **(A)** Decrease in the power spectrum (DPS) surrounding IEDs. DPS symmetrically surrounded the IED negative peaks and related IPS (−400 to 400 ms, 4–40 Hz) was observed independently to the baseline period considered [−2,000 ms; 1,500 ms], [−1,000 ms; −500 ms] (patient 4) (*p* < 0.001). **(B)** Typical IPS patterns related to spike-wave. Independently to the baseline period considered, a typical IPS related to the first negative peak of the spike was followed by a second IPS in the range of 4–10 Hz (100–400 ms), corresponding to the slow-wave, resulting in a “boot shape” (Patient 3) (*p* < 0.001). The red lines corresponded to the baseline periods.

TFA demonstrates spectral modulations [i.e., increased power spectra (IPS) or decreased power spectra (DPS)], especially between 10 and 30 Hz (see results section, Figures 2, 3). Thus, two time-frequency windows were selected around the IPS window (−50 to 50 ms, 10–30 Hz) or DPS (−400 to −100 ms, 10–30 Hz). A time-frequency source localization approach (i.e., Beamformer) was applied to these two-time windows.

The beamformer is a modified version of the linearly constrained minimum variance vector beamformer in the time-frequency domain as described in Gross et al. (22). It allows to image evoked and induced oscillatory activity in a time-frequency range, where time is taken relative to a triggered event.

The computation is based on a transformation of each channel's single trial data from the time domain into the time-frequency domain. This transformation leads to the complex spectral density Si (f, t), where i is the channel index and f and t denote frequency and time, respectively. Complex cross spectral density matrices C are computed for each trial:

$$\begin{array}{l} C_{ij}\left(f,t\right)=S_{i}\left(f,t\right).S_{j}^{*}\left(f,t\right) \end{array}$$

The output power P of the beamformer for a specific brain region at location r is then computed by the following equation:

$$\begin{array}{l} P\left(r\right)=tr^{\prime }{\left[L^{T}\left(r\right).C_{r}^{-1}.L\left(r\right)\right]}^{-1} \end{array}$$

Here, ${\text{C}}_{\text{r}}^{-1}$ is the inverse of the SVD-regularized average of *C*_*ij*_ *(f, t)* over trials and the time-frequency range of interest; *L* is the leadfield matrix of the model containing a regional source at target location *r* and, optionally, additional sources whose interference with the target source is to be minimized; tr′[] is the trace of the [3 × 3] submatrix of the bracketed expression that corresponds to the source at target location r.

**Figure 4:**
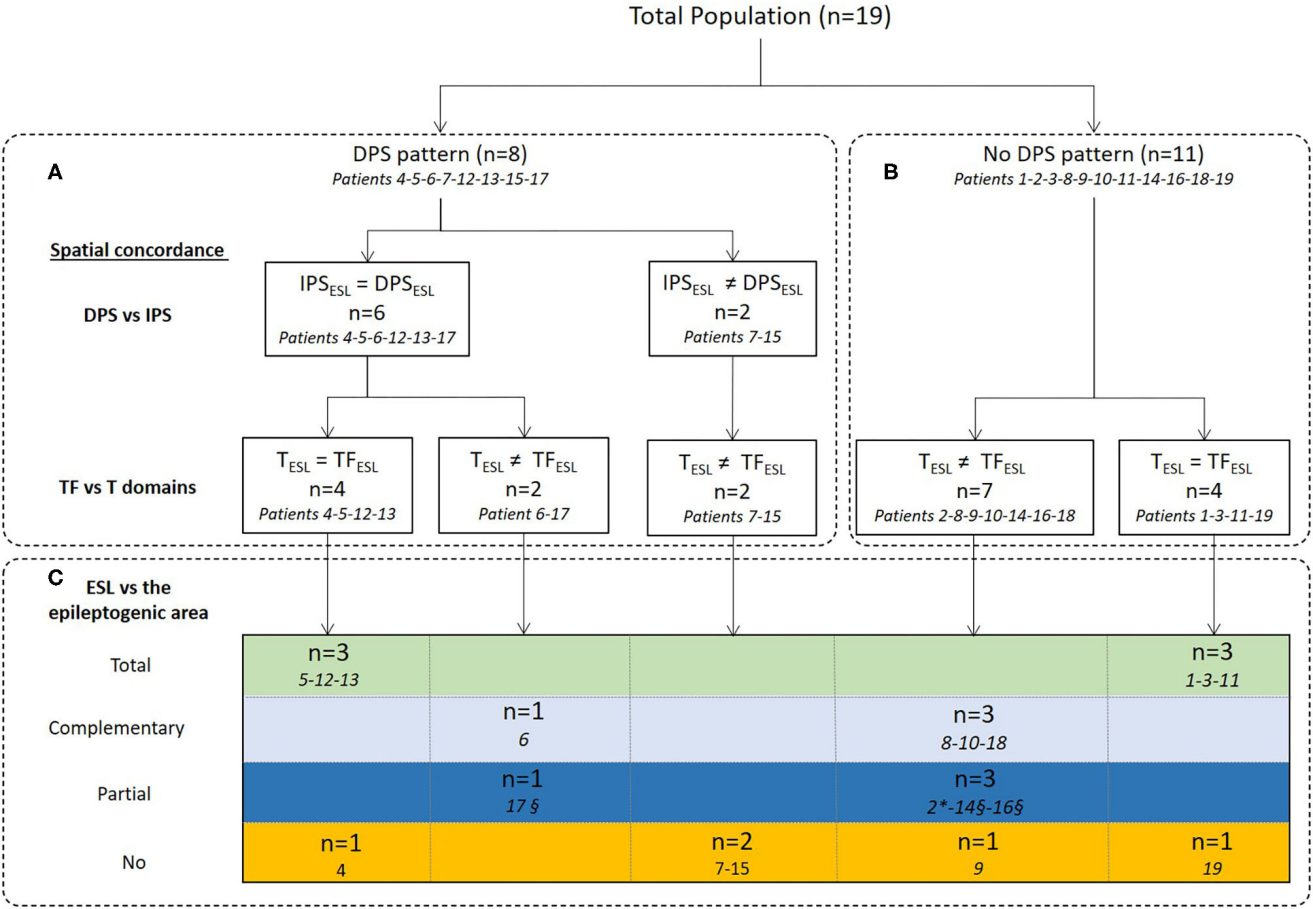
Concordance between ESL in the time and time-frequency domains and comparison with the epileptogenic sub-lobar area. ESL results were first compared between IPS and DPS **(A)** in the time-frequency domain and then spatial concordance with ESL in the time domain was analyzed **(A,B)**. Finally, ESL in the time and time-frequency domains were compared to the epileptogenic sub-lobar area **(C)**. IPS_ESL_= DPS_ESL_: ESL of IPS and DPS were localized to the same sub-lobar area. IPS_ESL_ ≠ DPS_ESL_: ESL of IPS and DPS were localized to different sub-lobar areas. T_ESL_ = TF_ESL:_ ESL results in time and time-frequency domains were localized to the same sub-lobar area. T_ESL_ ≠ TF_ESL:_ ESL results in time and time-frequency domains were localized to different sub-lobar areas. Spatial concordance was considered to be total when ESL in the time and time-frequency domains were localized to the same sub-lobar area and the epileptogenic sub-lobar area (in green) and complementary when they were localized to different sub-lobar areas, including the epileptogenic sub-lobar area (in gray). The spatial concordance was considered to be partial (in blue) when only one methodology gave results concordant with the epileptogenic sub-lobar area. In orange, no spatial concordance between the ESL methodologies. *Only ESL in the time domain was concordant with the epileptogenic sub-lobar area. ^§^Only ESL in the time-frequency domain (IPS) was concordant with the epileptogenic sub-lobar area.

**Figure 5:**
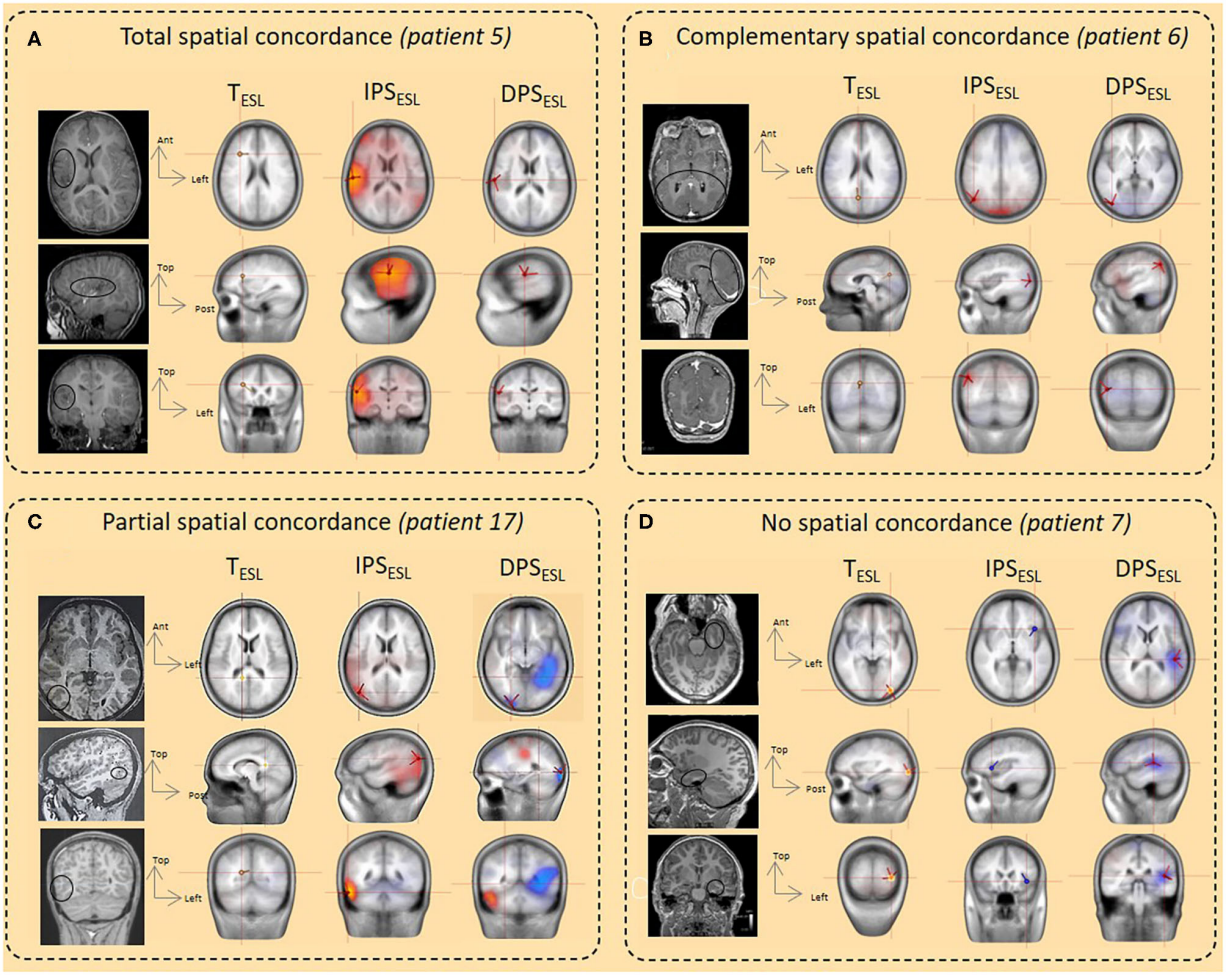
Illustration of spatial concordance between ESL in the time and time-frequency domains and comparison with the epileptogenic sub-lobar area. **(A)** Total spatial concordance. The spatial concordance was total between T_ESL_, IPS_ESL_, and DPS_ESLand_ with the epileptogenic area and the ischemic lesion (balck circle, MRI) (*patient 5*). **(B)** Complementary spatial concordance. The spatial concordance was complementary between T_ESL_, right medial occipital (M occ) and IPS_ESL_/DPS_ESL_ (lateral occipital (L occ) with the epileptogenic area and cortical occipital malformation (black circle, MRI) (*patient 6*). **(C)** Partial spatial concordance. The spatial concordance was partial between T_ESL_ and IPS_ESL_/DPS_ESL_. T_ESL_ was not localized in the epileptogenic area whereas IPS_ESL_/DPS_ESL_ were concordant with the epileptogenic area and the cortical dysplasia in the lateral right occipital region (balck circle, MRI) (*patient 17*). **(D)** No spatial concordance. No spatial concordance was observed between the ESL methods and the epileptogenic area localized in the left mesio-anterior temporal region (black circle, MRI) (*patient 7*).

**Figure 6:**
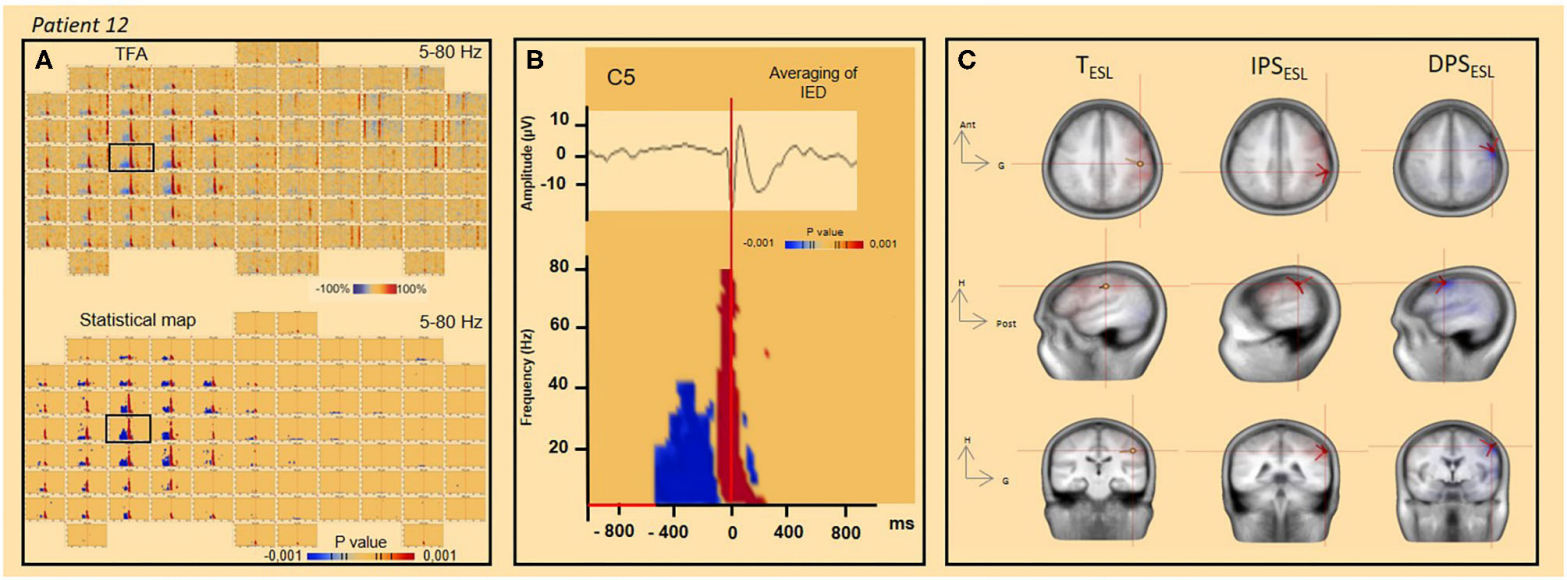
TFA and significant statistical results (*p* < 0.001) for time frequency analysis and ESL concordance (*patient 12*). **(A)** TFA and significant statistical results (*p* < 0.001) of time frequency analysis [4–80 Hz] for all channels. The channel C5 is indicated by the black square. **(B)** Spike-wave averaged IEDs (on the top) and significant statistical results (*p* < 0.001) of time-frequency analysis [4–80 Hz] (channel C5). **(C)** ESL in time (dipole fit) and time-frequency (DPS/IPS) domains.

ESL in the time and time-frequency domains were compared and their spatial concordance with the epileptogenic sub-lobar area analyzed (Table 2, Figures 4–6). ESL results were considered spatially concordant when they were localized in the same sub-lobar area. *Total spatial concordance* was defined when ESL in the time and time-frequency domains were localized (1) in the same sub-lobar area and (2) in the epileptogenic sub-lobar area. *Complementary spatial concordance* was defined when ESL in the time and time-frequency domains were localized (1) in different sub-lobar areas and (2) in the epileptogenic sub-lobar area. *Partial spatial concordance* was defined when ESL in the time and time-frequency domains were localized (1) in different sub-lobar areas and (2) when only one method localized the source in the epileptogenic sub-lobar area.

**Table 2:** TFA modulations, and the ESL results in the time and time-frequency domains of the RFE children population.

**Patients**	**Epileptogenic sub-lobar area**		**IED**				**Source Localization**		
			**Type**	**Number of events**	**Atypical IPS pattern**	**DPS pattern**	**IED**	**IPS**	**DPS**
1	Right	Pref (DL-DM)	Spike-wave	20	HFO		✓	✓	
2	Left	Oper-Ins Temp (MA-MP)	Poly-spike-wave	24			✓	*x*	
3	Left	Pref (DL-VL-VM-DM)	Spike-wave	44			✓	✓	
4	Right	Temp (LP)	Spike-wave	30	HFO	Yes	*X*	*X*	*X*
5	Right	Premot (L) Cent (L)	Spike-wave	20		Yes	✓	✓	✓
6	Right	occ (L-M)	Spike-wave	14	Isolated IPS islands	Yes	✓	✓	✓
7	Left	Temp (MA)	Spike	27		Yes	*X*	*X*	*X*
8	Left	Pariet (L) Temp (LP-MP) Oper-Ins	Spike-wave	20			✓	✓	
9	Right	Temp (LA-MA)	Spike	10			*X*	*X*	
10	Left	Pariet (L-M)	Spike	129			✓	✓	
11*	Left	Pref (DL-VL-VM-DM)	Spike	24			✓	✓	
12	Left	Pariet (L-P) Occ (L-M)	Spike wave	38		Yes	✓	✓	✓
13	Right	Temp (LP) Occ (L)	Spike-wave	16		Yes	✓	✓	✓
14*	Right	Temp (LP-MP) Pariet (M-L)	Spike-wave	55			*X*	✓	
15	Left	Temp (MA-LA)	Spike	130		Yes	*X*	*X*	*X*
16	Left	Pariet (L)	Spike-wave	35			*X*	✓	
17	Right	Occ (L)	Spike	160		Yes	*X*	✓	✓
18*	Right	Pariet (L-M)	Spike-wave	36			✓	✓	
19	Left	Temp (LA-MA)	Spike	39			*X*	*X*	

*IED types and TFA modulations were determined for each patient. Specific IPS patterns were observed for three patients, a localized IPS pattern (−200 to −100 ms, 15–25 Hz) for one patient (patient 6) and isolated IPS islands (−100 to 100 ms, 100–150 Hz) for two, likely corresponding to high-frequency oscillations (HFO) (patient 1–4)*.

*ESL in the time and time-frequency domains were compared to the epileptogenic sub-lobar area. ✓, ESL correctly localized to the epileptogenic sub-lobar area, X, ESL localized outside to the epileptogenic sub-lobar area*.

*Spatial concordance was considered to be total when ESL in the time and time-frequency domains were localized to the same sub-lobar area and the epileptogenic sub-lobar area (in green) and complementary when they were localized to different sub-lobar areas, including the epileptogenic sub-lobar area (in gray). The spatial concordance was considered to be partial (in blue) when only one methodology gave results concordant with the epileptogenic sub-lobar area. In orange, no spatial concordance between the ESL methodologies*.

Y, years; m, months; FIAS, focal impaired-awareness seizures; FAS, focal aware seizures; R, right; N/A, not available; L, left; VM pref, ventral-medial prefrontal; DM pref, dorsal-medial prefrontal; VL pref, ventral-lateral prefrontal; DL pref, dorsal-lateral prefrontal; M premot, medial premotor; L premot, lateral premotor; M cent, medial central; L cent, lateral central; MA temp, medial-anterior temporal; LA temp, lateral-anterior temporal; MP temp, medial-posterior temporal; LP temp, lateral-posterior temporal; M pariet, medial parietal; L pariet, lateral parietal; M occ, medial occipital; L occ, lateral occipital; Oper-Ins, operculo-insular; and Junc, temporo-parieto-occipital junction; * patients recorded using 128-channel EGI EEG system.

## Results

We first analyzed TFA modulations specific to the different types of IED (spike, spike-wave or polyspike-wave) and then the spatial distribution of IEDs in the time and time-frequency domains.

### Increase in the Power Spectrum (IPS) Related to IEDs

#### Typical IPS Patterns

IPS patterns were specific to different IEDs (Figure 2A, Table 2), independently of the selected baseline periods (Figure 3). For spikes (seven patients), the TFA pattern consisted of only one IPS (−100 to 100 ms around T0, 4–50 Hz). For spike waves (11 patients), this IPS continued with a second IPS in a restricted range of lower frequencies (4–10 Hz, 100–400 ms), corresponding to the slow-wave recorded on the EEG and, altogether, resulting in a “boot shape” in the TFA. For polyspike waves (one patient), successive IPSs, corresponding to the successive spikes, were followed by the same boot-shaped pattern concomitant to the slow-wave.

#### Island of Atypical IPS Patterns

In addition to the typical IPS pattern, atypical IPS patterns were also observed (Figure 2C, Table 2). These atypical IPS patterns were observed independently of the selected baseline periods (Figure 3). In one patient, the IPS related to the IED (Patient six) was preceded by a focalized IPS pattern (−200 to −100 ms) at a specific frequency range (15–25 Hz) (Figure 2C). In two patients (Patients one and four), IPSs related to IEDs were associated with isolated islands of IPSs (−100 to 100 ms) at a specific frequency range (100–150 Hz), likely corresponding to high-frequency oscillations (HFO) (Figure 2C).

### Decrease in the Power Spectrum (DPS) Surrounding the IEDs

In eight patients, a DPS symmetrically surrounded the first negative peaks of the IED and its related IPS (−400 to 400 ms, 4–40 Hz) (Figures 2A, 3, 6, Table 2). DPS patterns were independent from the selected baseline (Figure 3). DPS were not specific to the type of IED and were observed surrounding spikes (three of seven patients) and spike waves (five of 11 patients), but not polyspike waves (zero of one patient). DPS were not specific to the type of epilepsy and were observed in both lesional (six of 13 patients) and non-lesional (two of six patients) epileptic patients. DPS were not specific to the epileptogenic sub-lobar area.

### Control Conditions

#### Random Epochs

Thirty IED-free epochs were randomly selected to ensure that the modulations observed by TFA were specific to the pathological activity selected. No relevant changes in TFA were observed.

#### Vertex Sharp Waves

Vertex sharp waves were assessed in two patients (Figure 2B) in the time-frequency domain. An IPS concomitant to the vertex sharp wave in the time domain was observed (−100 to 200 ms, 4–40 Hz) without any other power spectral modulations.

### ESL in the Time and the Time-Frequency Domains

The time-frequency source localization approach was applied for both the IPS and DPS for eight patients. For six of eight patients presenting both IPS and DPS, the time-frequency source localization was spatially concordant between the two (i.e., localized in the same sub lobar area) and discordant (i.e., localized in different same sub lobar areas) for the two remaining patients (with mesio-temporal epilepsy) (Figure 4A, Table 2). When the time-frequency source localization of the IPS and DPS were spatially concordant (*n* = 6), ESL in the time-domain was also spatially concordant for four in discordant for two. When the time-frequency source localization of the IPS and DPS were not concordant, ESL in the time-domain was also discordant (*n* = 2, with mesio-temporal epilepsy) (Figure 4A, Table 2).

Time-frequency source localization was applied only to the IPS for 11 patients (Figure 4B, Table 2). The ESL of the IPS were spatially concordant with that of the time domain for four patients and not spatially concordant for seven.

**Figure 7:**
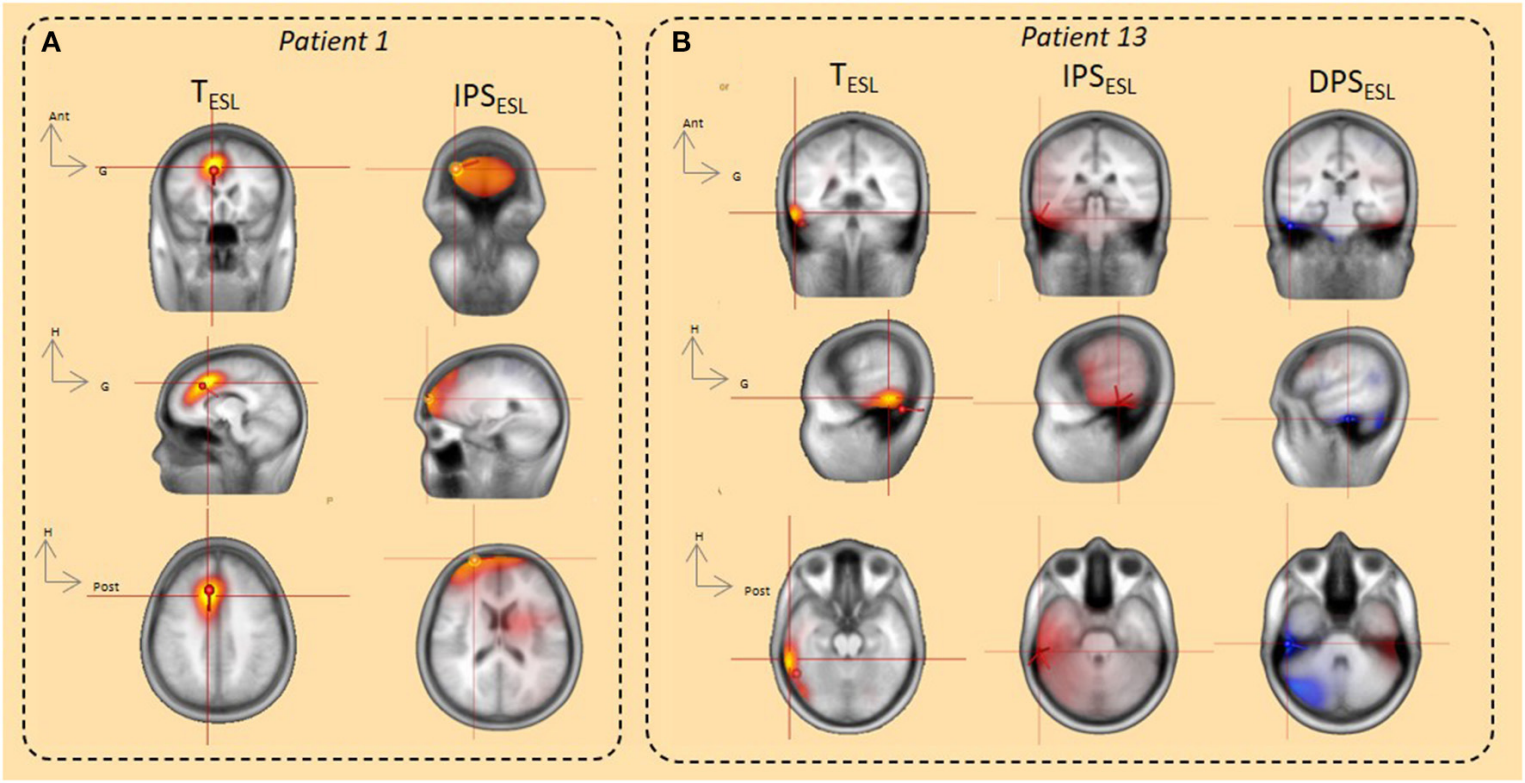
ESL using the dipole fitting and distributed methods (CLARA, SSLOFO) in the time domain and beamformer in the time-frequency domain. **(A)** In the time domain, both dipole fit and distributed methods (CLARA) localized IED origin in the right prefrontal area. ESL in the time domain was concordant with ESL in time frequency domain (IPS) and with the epileptogenic area (*patient 1*). **(B)** In the time domain, both dipole fit and distributed methods (SSLOFO) localized IED origin in the right lateral temporal area. ESL in the time domain was concordant with ESL in the time-frequency domain (DPS/IPS) and with the epileptogenic area (*patient 13*).

For 14 of the 19 patients, at least one ESL method was concordant with the epileptogenic sub-lobar area. ESL in the time and time-frequency domains (DPS/IPS) localized the source to the same sub-lobar region in six of these 14 patients. For four of the 14, ESL in the time-frequency domain provided complementary information to the ESL time domain. For three of the 14 patients, the time-frequency method was only concordant with the epileptogenic area, and for the remaining patient, the time-domain method was only concordant with the epileptogenic sub-lobar area (Figures 4C, 5–7, Table 2).

For five of the 19 patients, ESL in the time and time-frequency-domains were not concordant with the epileptogenic sub-lobar area. All of these five patients presented with temporal epilepsy. For two of the five patients, ESL in the time and time-frequency-domains were localized to the same sub-lobar region, without concordance with the epileptogenic sub-lobar area. For three of the five epileptic patients, there was no concordance between the different ESL methods (Figures 4C, 5–7, Table 2).

## Discussion

We observed different patterns of time-frequency characteristics surrounding the IED: (1) an IPS centered around the IED (−100 to 100 ms), regardless of whether they were spikes, spike-waves or polyspike waves, (2) DPS symmetrically surrounding the IES [−400 to −100 ms and 100–400 ms], and (3) islands of atypical IPS. These TF patterns were spatially distributed and may provide complementary information concerning the spatial distribution of neuronal interactions within and outside the epileptogenic sub-lobar area.

### Time-Frequency Modulation Surrounding the IEDs

Time-frequency modulations surrounding IEDs highlight the complexity of the mechanisms involved in IED generation. These specific TFA patterns could be considered as biomarkers of epileptic processes (7, 9, 10).

In RFE patients, a typical neuronal IPS (4–50 Hz) concomitant with the negative peaks of the IEDs can be preceded by several hundred milliseconds (~400 ms) by TFA modulation consisting of a DPS or an isolated island IPS in a restricted lower frequency range (4–50 Hz), regardless of IED type. Such island IPS occurring before the spike might correspond to the rhythmic bursting pattern or to fast runs of EEG activity in the range of 10–15 Hz (23, 24). Such rhythmic bursting in the range of 10–15 Hz is considered to be a widespread phenomenon serve by long range excitatory connections (25). Considering DPS, previous studies using local field-potential recordings and/or multi-unit activity (26) have demonstrated heterogeneous neuronal activity beginning before IEDs. Manoocheri et al. (5, 6) demonstrated that time-frequency modulations occur concomitantly with sequences of neuronal shrinking-swelling-shrinking (i.e., deactivation-activation-deactivation) in rats and epileptic children using fast optical imaging (5, 6). These preceding complex neuronal interactions may constitute a time window in which suitable conditions are met for propelling neuronal synchronization that makes up an IED. The decrease in interneuron cortical firing several hundred milliseconds before IEDs can result in a hypersynchronous excitatory rebound (9) that makes up the fast component of IEDs (4, 27).

Early-onset TFA modulations were observed for approximately half of the patients. This is consistent with the observations of Keller et al., who reported inconsistent changes in the neuronal firing rate before IEDs, suggesting that there are various strategies to reach the IPS of the IEDs (9). Alternatively, an insufficient EEG signal-to-noise ratio cannot be ruled out to explain this inconsistency. In these focal epilepsies, only one type of IEDs in terms of morphology and spatial distribution was selected per patient. Nevertheless, the IEDs with similar patterns might have slight variations in amplitudes, corresponding to the recruitment of a more or less extended neuronal population.

Apart from RFE, similar time-frequency modulations have also been observed in self-limited focal epilepsy in children (i.e., epilepsy with centro-temporal spikes) (10) and bicuculline-induced epileptic rats (5, 6), suggesting that this mechanism is generic and involved in various types of epilepsy (10), independently of the species (5, 6). In contrast, our study suggested that TFA patterns might be specific to the type of IED. However, complementary studies on larger cohorts with different types of IEDs are required to confirm our current results.

### Time-Frequency Modulations Spatially Related to IEDs

Modulations (DPS) are observed several hundred milliseconds before IEDs, which represents a long time relative to that of neuronal transmission. A temporo-spatial approach (i.e., HD-EEG combined with TFA) makes it possible to address the spatial distribution of distinct neuronal populations in the epileptic network, allowing the exploration of cortical spatial dynamics with high spatial resolution at the macroscopic level. Spatial concordance differs between temporal and extra-temporal epilepsy.

For temporal and, more specifically, mesial temporal lobe epilepsy, ESL in the time and time-domains were not concordant with the epileptogenic zone. The deep localization of the IED electrical sources and their geometry, which led to electrical field potential cancellations and a blurring effect of the neocortical activity, are likely to account for such discrepancies, regardless of the source localization in the time or time frequency domain (28, 29).

For extra-temporal epilepsy, ESL in the time and time-frequency domains provided both concordant and complementary spatial information's about the epileptogenic zone. In the time-frequency domain, ESL performed from DPS and IPS were both spatially concordant between the methodologies and successfully localized the epileptogenic zone at a sub-lobar level for all patients with extra-temporal epilepsy. The concordance in the ESL from DPS and IPS is in accordance with the results of Keller et al. (9), who demonstrated that early changes in neuronal discharges, preceding the hyperactivity that constitutes cortical IEDs, occurred specifically in or near the seizure onset zone. Thus, concordant results between the ESL performed in the time-frequency domain from DPS and IPS, as well as the time domain, could contribute to the evidence used to correctly localize the epileptogenic zone. The spatial discordance between the various ESL methodologies in extra-temporal epilepsy should also be considered and may illustrate the complexity of the network distribution at a mesoscopic level.

In summary, time and time-frequency ESL performed with HD-EEG provide a global overview of the dynamics of the interictal epileptic network, which should be taken into account during the pre-surgical evaluation and could be of interest in creating the schema of stereotaxic EEG electrode implantation. Moreover, the clinical superiority of distributed approach on dipole modeling is still a matter of debate for focal epilepsy, including focal self-limited epilepsy (30), it cannot be ruled out that multiple dipoles might better explain the IEDs. Even, if no obvious localization differences according to the inverse methods used (dipolar and distributed) could be demonstrated in the present study, the complementary of these methods can be of clinical value. In this study, the clinical validated statistical toolbox (BESA® research) was used. Others statistical approach might yield an increase in the significance of the results in this domain (31). Further studies with a larger population of patients with RFE with surgical outcomes are required to confirm the added value of this combined approach.

## Conclusion

TFA extracted from HD-EEG provides fundamental neurophysiological insights into the complex mechanisms that propel neurons toward the generation of IEDs. IEDs reflect the interplay of multiple distinct neuronal types within complex neuronal networks. In addition, TFA demonstrates complex time-frequency modulations of the neuronal networks around the IEDs, suggesting that the pathological mechanisms are initiated well before the onset of the classical hyper-synchronization of the IED.

Combining time and time-frequency analysis of the ESL provides complementary information to define the epileptogenic zone in refractory focal epilepsy. Except for temporo-mesial epilepsy, the spatial concordance between time and time-frequency methodologies contribute to the evidence used to localize the epileptogenic zone. Discordance should also be considered as sentinel information when stereotaxic EEG electrode implantation is being considered.

## Data Availability Statement

The datasets presented in this article are not readily available because the ethical approval doesn't include datasets availability. Requests to access the datasets should be directed to Fabrice Wallois, fabrice.wallois@u-picardie.fr.

## Ethics Statement

The studies involving human participants were reviewed and approved by The local ethics committee approved this study (CPP Nord-Ouest, No. A00782-39). Written informed consent to participate in this study was provided by the participants‘ legal guardian/next of kin.

## Author Contributions

EB-P, MM, YJ, and FW conceived and designed the experiments. EB-P, NM, and YJ performed the experiments. EB-P, YJ, MM, and FW contributed reagents/materials/analysis tool. CH, EB-P, MM, YJ, and FW wrote the paper. CH, EB-P, MM, NM, and FW read and accepted the manuscript. All authors contributed to the article and approved the submitted version.

## Conflict of Interest

The authors declare that the research was conducted in the absence of any commercial or financial relationships that could be construed as a potential conflict of interest.
